# GCKR gene functional variants in type 2 diabetes and metabolic syndrome: do the rare variants associate with increased carotid intima-media thickness?

**DOI:** 10.1186/1475-2840-9-79

**Published:** 2010-11-29

**Authors:** Márton Mohás, Péter Kisfali, Luca Járomi, Anita Maász, Eszter Fehér, Veronika Csöngei, Noémi Polgár, Enikő Sáfrány, Judit Cseh, Katalin Sümegi, Katalin Hetyésy, István Wittmann, Béla Melegh

**Affiliations:** 1 2nd Department of Medicine and Nephrological Center, University of Pécs, Pécs, Hungary H-7624 Pacsirta 1; 2 Department of Medical Genetics, University of Pécs, Pécs, Hungary H-7624 Szigeti 12; 3 Department of Radiology, University of Pécs, Pécs, Hungary H-7624 Ifjúság 13; 4 Central Laboratory, Aladár Petz Hospital, Győr, Hungary, H-9024 Vasvári Pál 2

## Abstract

**Background:**

Recent studies revealed that glucokinase regulatory protein (GCKR) variants (rs780094 and rs1260326) are associated with serum triglycerides and plasma glucose levels. Here we analyzed primarily the association of these two variants with the lipid profile and plasma glucose levels in Hungarian subjects with type 2 diabetes mellitus and metabolic syndrome; and also correlated the genotypes with the carotid intima-media thickness records.

**Methods:**

A total of 321 type 2 diabetic patients, 455 metabolic syndrome patients, and 172 healthy controls were genotyped by PCR-RFLP.

**Results:**

Both GCKR variants were found to associate with serum triglycerides and with fasting plasma glucose. However, significant association with the development of type 2 diabetes mellitus and metabolic syndrome could not be observed. Analyzing the records of the patients, a positive association of prevalence the GCKR homozygous functional variants and carotid intima-media thickness was found in the metabolic syndrome patients.

**Conclusions:**

Our results support that rs780094 and rs1260326 functional variants of the GCKR gene are inversely associated with serum triglycerides and fasting plasma glucose levels, as it was already reported for diabetic and metabolic syndrome patients in some other populations. Besides this positive replication, as a novel feature, our preliminary findings also suggest a cardiovascular risk role of the GCKR minor allele carriage based on the carotid intima-media thickness association.

## 1. Background

Type 2 diabetes mellitus (T2DM) is characterized by elevated plasma glucose level as a result of impaired beta-cell function and/or peripheral insulin resistance [1]. Impaired glucose regulation is a major hallmark of metabolic syndrome (MS), however it is a more complex disorder featured by visceral obesity, elevated serum triglycerides, low level of HDL-cholesterol and raised blood pressure [2]. The prevalence of T2DM and MS is very high in the industrialized countries contributing to a considerably increased atherosclerotic burden and cardiovascular risk. Both of them are multifactorial diseases, besides several environmental factors, such as cigarette smoking, obesity, lack of exercise, bad nutrition habits and genetic factors are also contributed to the pathogenesis.

Glucokinase (GCK) is a predominant glucose phosphorylating enzyme expressed in the liver and in the beta-cells of the Langerhans islets, playing a pivotal role in the glucose-stimulated insulin release as a physiological glucose-sensor [3]. Pancreatic islets and the liver contain a regulatory protein (glucokinase regulatory protein, GCKR), which inhibits GCK in an allosteric manner with respect to glucose concentration by forming an inactive heterodimer. The inhibitory effect of GCKR is enhanced by fructose-6-phosphate and antagonized by fructose-1-phosphate [4]. The 27 kb GCKR gene is located on chromosome 2p23 containing 19 exons and encodes a 68 kDa protein [5,6]. Genome-wide association studies showed, that common functional variants of the GCKR gene are associated with fasting plasma glucose, insulin levels, and both serum triglycerides and low/high-density lipoprotein cholesterol levels, thus, single nucleotide polymorphisms (SNPs) rs780094 and rs1260326 reduce fasting plasma glucose concentration and insulin levels and improve insulin resistance, while inversely increase fasting and postprandial serum triglycerides [7-16]. More recently, both functional variants of the GCKR gene were widely investigated as candidate T2DM susceptibility variants, and a protective nature against T2DM [8,10,17,18].

The primary goal of the current work was to study the possible association of rs780094 and rs1260326 of the GCKR gene on metabolic and cardiovascular risk traits in Hungarian patients, which nation differs from the surrounding European populations in its origin [19]. The pooled ultrasonography records of the patients enabled us to study also the carotid intima-media thickness association.

## 2. Methods

### 2.1. Study population

In a genetic association study we examined two common variants (rs780094 and rs1260326) of the GCKR gene. The study population comprised 321 subjects with T2DM (172 males, 149 females, mean age: 61.3 ± 12.2 years, range: 27-89 years), 455 subjects with MS (200 males, 255 females, mean age: 61.7 ± 10.7, range: 26-85 years) and 172 healthy control subjects (49 males, 123 females, mean age: 56.5 ± 15.2, range: 19-92 years). All 948 study participants were selected from the Caucasian Hungarian population.

All patients were enrolled from the 2^nd ^Department of Medicine and Nephrological Center, University of Pécs, Hungary and from the Aladár Petz Hospital, Győr, Hungary. T2DM was diagnosed according to the criteria of the World Health Organization [1]. Waist circumference data were not available, therefore MS was diagnosed according to modified criteria of the Adult Treatment Panel III of National Cholesterol Education Program [20], defined as presence of at least 3 of the following factors at the time of diagnosis: body mass index (BMI) > 30 kg/m^2^, serum triglycerides≥1.70 mmol/l and/or lipid lowering treatment; serum HDL-cholesterol < 0.9/1.1 mmol/l (male/female); systolic blood pressure≥130 mmHg and diastolic blood pressure≥85 mmHg and/or antihypertensive treatment; fasting plasma glucose≥5.60 mmol/l and/or antiglycemic treatment. Hypertriglyceridemia was defined as fasting serum triglycerides≥1.7 mmol/l. Controls were gathered from trauma units, blood donors, medical staff and university students, they were free from any single clinical or laboratory sign of T2DM or MS; their medical history were also free from any systemic or organ-specific disease. Exclusion criteria were as follows: pregnancy, fever, sepsis, malignancies, autoimmune systemic diseases, alcohol or drog abuse, severe heart failure, hepatic failure.

DNA samples and the clinical data were deposited into the Central Biobank governed by the University of Pécs, as part of the National Biobank Network of Hungary http://www.biobank.hu, approved by the national Scientific Research Ethics Committee (ETT TUKEB). The Biobank belongs to the pan-European Biobanking and Biomolecular Resources Research Infrastructure (BBMRI) preparatory phase project http://bbmri.eu/bbmri/. All participants gave their informed consent and the study followed the principles of the Helsinki Declaration.

### 2.2. Biochemical and clinical data

All participants underwent a detailed medical examination, including anamnestic history, physical examination, and estimation of cardiovascular risk factors, laboratory and urine analysis, electrocardiography. Laboratory parameters were assessed using routine methods from fasting blood samples. BMI was calculated as weight (kg) divided by height (m^2^). Carotid intima-media thickness (CIMT) was measured by B-mode ultrasound device with a high resolution 10 MHz linear transducer (ALOKA 4000, Tokyo, Japan) in a plaque free region of both carotid arteries at 2 cm proximal to the carotid bulb on the common carotid artery and at the origin of the internal carotid artery. The ultrasound transducer was placed in an angle of 90° of the vessel wall. CIMT values were obtained from the above mentioned sites and were defined as means of the maximal CIMT measurements for the right and left sides. Each participants were scanned in a standardized environment, in the same room, by the same examiner, using the same instrument.

### 2.3. Genotyping

DNA was isolated from peripheral blood leukocytes by standard salting out method. For polymerase chain reaction amplification, the following primers (MWG-Biotech AG, Ebersberg, Germany) were used: GCKR rs1260326: forward 5'-TGC AGA CTA TAG TGG AGC CG-3' and reverse 5'-CAT CAC ATG GCC ACT GCT TT-3'; GCKR rs780094: forward 5'-GAT TGT CTC AGG CAA ACC TGG TAG-3' and reverse 5'-CTA GGA GTG GTG GCA TAC ACC TG-3'. An MJ Research PTC-200 thermal cycler (Bio-Rad, Hercules, CA, USA) was used for the amplification. PCR conditions were the following: predenaturation at 96°C for 2 min; followed by 35 cycles of denaturation at 96°C for 20 sec (rs1260326), 30 cycles of denaturation at 96°C for 20 sec (rs780094); annealing at 60°C for 20 sec (rs1260326), and at 62°C for 30 sec (rs780094); primer extension for 30 sec at 72°C; and final extension at 72°C for 5 min. The amplicons were digested by HpaII restriction endonuclease (rs1260326) and PscI (rs780094) (Fermentas, Burlington, ON, Canada). The digestion of 231 bp amplicon of rs1260326 CC genotype resulted in 18, 63, 150 bp fragments; the TT genotype 18 and 213 bp; while the heterozygous genotype 18, 63, 150, 213 bp fragments. For the rs780094 427 bp amplicon the following fragments were detected: GG genotype 62, 177, 188 bp; AA genotype 62, 365 bp; heterozygous genotype 62, 177, 188, 365 bp fragments. All methods were designed to include an obligate cleavage site on the amplicon to enable us to control the efficacy of the digestion.

### 2.4. Statistical analysis

BMI, fasting plasma glucose concentrations, serum triglycerides and HDL-cholesterol levels were log-transformed because of non-normal distribution. Results were expressed as mean ± SD and median (minimum-maximum) as appropriate according to distribution. Kolmogorov-Smirnov test was used to assess sample distribution. Chi-square test was carried out to determine whether genotype distributions followed the Hardy-Weinberg equilibrium and to compare other qualitative data. Clinical and biochemical characteristics of the study participants at baseline were compared with one-way ANOVA. Statistical differences between the individual GCKR genotypes were assessed by analysis of covariance (ANCOVA) adjusted for gender, age, BMI. Trend was examined with Jonckheere-Terpstra test. Logistic regression analysis models were used to evaluate individual effects of genotypes as possible risk factors; multivariate regression analysis models were adjusted for age, gender, total serum cholesterol, coronary artery diseases (CAD) and statin therapy. All statistical procedures were performed using the SPSS 15.0 software (SPSS Inc., Chicago, IL, USA). P values ≤ 0.05 were considered statistically significant.

## 3. Results

Major clinical and biochemical features of the patients and controls are summarized in Table 1; the genotype profiles including the minor allele frequencies are shown in Table 2. All genotypes were in Hardy-Weinberg equilibrium. Allele frequencies were similar in the study groups.

**Table 1:** Clinical and biochemical features of the patients with T2DM, MS and control subjects.

	Controls n = 172	T2DM n = 321	MS n = 455	p-value
Gender (male/female)	49/123	172/149	200/255	< 0.001
Age (years)	56.5 ± 15.2	61.3 ± 12.2	61.7 ± 10.7	< 0.001*
Body mass index (kg/m^2^)	23.9 ± 2.15	29.5 ± 5.87	33.3 ± 5.48	< 0.001*^#^
Fasting plasma glucose (mmol/l)	N/A	8.70 (2.30-22.8)	9.00 (3.00-30.8)	0.249
Serum total cholesterol (mmol/l)	4.78 ± 1.10	5.16 ± 1.25	5.37 ± 0.94	< 0.001^†^
Serum LDL-cholesterol (mmol/l)	N/A	2.74 ± 0.86	2.83 ± 0.84	0.256
Serum HDL-cholesterol (mmol/l)	N/A	1.19 (0.55-2.12)	1.23 (0.55-2.49)	< 0.001
Serum triglycerides (mmol/l)	1.50 (0.50-3.60)	1.62 (0.35-14.8)	1.98 (0.35-14.48)	< 0.001^#†^
CIMT (mm)	N/A	0.88 ± 0.44	1.22 ± 0.74	0.265
Systolic blood pressure (mmHg)	N/A	130 (87.0-210)	140 (70.0-200)	< 0.001
Diastolic blood pressure (mmHg)	N/A	80.0 (50.0-130)	80.0 (60.0-137)	< 0.001
Hypertension (%)	22.7	77.5	90.4	< 0.001*
Coronary heart disease (%)	4.1	25.3	25.4	< 0.001*

CIMT, carotid intima-media thickness; HDL, high density lipoprotein; LDL, low density lipoprotein; N/A: data not available; MS: metabolic syndrome, T2DM: type 2 diabetes mellitus; * Controls vs. T2DM and MS; # T2DM vs. MS; † Controls vs. MS; Data are means ± SD or median (minimum-maximum) as appropriate.

**Table 2:** Genotype distribution (case number) and allele frequencies (%) in control subjects and in patients with T2DM and MS.

		Controls	T2DM	MS
**GCKR** **(rs780094)**	GG	44	77	121
	GA	93	151	217
	AA	35	78	100
	A allele (%)	47.4	46.9	47.6

**GCKR** **(rs1260326)**	CC	48	80	118
	CT	80	155	219
	TT	44	63	91
	T allele (%)	48.8	47.1	46.8

T2DM: subjects with type 2 diabetes mellitus; MS: subjects with metabolic syndrome

Table 3 shows the lipid parameters examined, the plasma glucose concentrations, BMI, and CIMT data for each analyzed SNP of the GCKR gene. BMI is not associated either with rs780094 or with rs1260326, however plasma glucose levels were associated with both variants significantly (p < 0.05). We observed association of the minor allele of rs780094 and rs1260326 with elevated serum triglycerides in all groups. We found no relationship between variants of GCKR gene and total serum cholesterol levels, however HDL-cholesterol level was significantly lower in subjects homozygous for the minor allele for both rs780094 and rs1260326, but only in T2DM patients, moreover LDL-cholesterol was significantly elevated in homozygous patients, but only in the MS group. We also correlated the CIMT of the patients with T2DM and MS. The homozygous minor alleles were associated with increased carotid intima-media thickness in metabolic syndrome patients.

**Table 3:** Body mass index, fasting plasma glucose, lipid profile and carotid intima-media thickness in subjects with metabolic syndrome, type 2 diabetes mellitus and controls by individual genotypes (A: GCKR rs780094; B: GCKR rs1260326)

		Controls			T2DM			MS		
		
		GG n = 44	GA n = 93	AA n = 35	GG n = 77	GA n = 151	AA n = 78	GG n = 121	GA n = 217	AA n = 100
	
A	BMI (kg/m^2^)	23.6 (21.2-28.6)	23.9 (15.8-32.8)	23.8 (13.7-26.8)	29.2 (17.0-48.6)	29.0 (18.3-45.9)	27.3 (18.3-43.4)	32.5 (22.5-52.5)	32.7 (20.4-48.1)	32.9 (19.4-60.5)
	FPG (mmol/l)	N/A	N/A	N/A	9.50 (3.00-17.0)	9.20 (3.80-22.8)	8.90 (2.30-17.1)*	9.60 (3.80-19.1)	8.80 (3.00-30.8)	8.50 (4.51-16.6)*
	Serum triglycerides (mmol/l)	1.35 (0.50-2.90)	1.50 (0.80-3.60)	1.70 (0.70-3.20)*	1.77 (0.38-8.23)	1.81 (0.35-6.22)	2.24 (0.66-14.8)*	2.07 (0.49-7.76)	2.68 (0.35-14.2)*	3.07 (0.78-12.3)#
	Serum total-cholesterol (mmol/l)	5.48 ± 0.80	5.44 ± 0.94	5.04 ± 1.04	5.01 ± 1.02	4.60 ± 1.12	4.75 ± 1.10	5.03 ± 1.05	5.15 ± 1.36	5.35 ± 1.26
	Serum HDL-cholesterol (mmol/l)	N/A	N/A	N/A	1.39 (0.76-2.48)	1.25 (0.68-2.37)	1.19 (0.62-2.49)*	1.21 (0.77-1.99)	1.22 (0.55-2.12)	1.17 (0.79-1.88)
	Serum LDL-cholesterol (mmol/l)	N/A	N/A	N/A	2.84 ± 0.90	2.64 ± 0.83	2.75 ± 0.89	2.08 ± 0.81	2.70 ± 0.83	3.06 ± 0.92*
	CIMT (mm)	N/A	N/A	N/A	0.81 ± 0.42	0.86 ± 0.38	0.95 ± 0.44	0.79 ± 0.28	0.87 ± 0.32	1.06 ± 0.26*
		**Controls**			**T2DM**			**MS**		
		
		**CC** **n = 48**	**TC** **n = 80**	**TT** **n = 44**	**CC** **n = 80**	**TC** **n = 155**	**TT** **n = 63**	**CC** **n = 118**	**TC** **n = 219**	**TT** **n = 91**
	
**B**	BMI (kg/m^2^)	23.7 (20.8-28-6)	23.8 (15.8-32.8)	23.8 (13.7-26.8)	29.2 (17.0-37.6)	29.3 (18.3-45.9)	28.9 (18.3-38.6)	31.8 (20.4-49.57)	32.8 (19.4-52.5)	33.4 (25.0-60.5)
	FPG (mmol/l)	N/A	N/A	N/A	9.05 (3.00-16.9)	8.90 (2.30-17.1)	8.70 (2.30-22.8)*	9.80 (3.20-20.0)	8.60 (3.90-30.8)	8.40 (3.00-16.6)*
	Serum triglycerides (mmol/l)	1.51 (0.50-2.90)	1.55 (0.70-2.90)	1.68 (0.80-3.60)*	1.45 (0.35-4.28)	1.87 (0.39-8.23)*	2.32 (0.59-14.9)*	2.19 (0.70-7.86)	2.72 (0.35-14.4)*	2.91 (0.78-11.5)*
	Serum total-cholesterol (mmol/l)	5.44 ± 0.87	5.41 ± 0.96	5.20 ± 0.99	5.03 ± 1.11	4.64 ± 0.97	4.72 ± 1.32	5.10 ± 1.02	5.21 ± 1.38	5.20 ± 1.27
	Serum HDL-cholesterol (mmol/l)	N/A	N/A	N/A	1.39 (0.76-2.48)	1.27 (0.68-2.47)	1.17 (0.62-2.49)*	1.23 (0.70-1.96)	1.16 (0.55-2.12)	1.23 (0.79-1.88)
	Serum LDL-cholesterol (mmol/l)	N/A	N/A	N/A	2.85 ± 0.88	2.63 ± 0.78	2.78 ± 1.05	2.71 ± 0.81	2.85 ± 0.82	3.00 ± 0.93*
	CIMT (mm)	N/A	N/A	N/A	0.81 ± 0.39	0.88 ± 0.37	0.95 ± 0.42	0.83 ± 0.32	0.87 ± 0.35	1.05 ± 0.36*

* p < 0.05 vs. GG; # p < 0.001 vs. GG; BMI, body mass index; CIMT, carotid intima-media thickness; FPG, fasting plasma glucose; HDL, high-density lipoprotein; LDL, low-density lipoprotein; MS, metabolic syndrome; T2DM, type 2 diabetes mellitus; Data are mean ± SD or median (minimum-maximum) as appropriate.

Table 4. shows the relative risk of hypertriglyceridemia for variants rs780094 and rs1260326 at GCKR gene calculated by the multiple logistic regression analysis models. Regression analysis revealed that both rs780094 and rs1260326 confer a significant susceptibility for the development of hypertriglyceridemia; after adjusting the results for age, gender, total serum cholesterol, CAD and statin therapy.

**Table 4:** Odds ratios at 95% confidence intervals (CI) calculated by the multiple logistic regression analysis models.

	Hypertriglyceridemia	
	**Unadjusted model** **Odds ratio** **(95% CI)**	**Adjusted model** Odds ratio* (95% CI)

**GCKR (rs780094)** **AA genotype**	1.748 (1.256-2.435) p = 0.001	5.335 (1.779-15.99) p = 0.003
**GCKR (rs1260326)** **TT genotype**	1.311 (1.078-1.596) p = 0.007	4.523 (1.458-14.03) p = 0.009

*Adjusted for: age, gender, BMI, serum total cholesterol, CAD, statins

## 4. Discussion

Genome wide association studies revealed an association between SNP rs780094 of GCKR gene and hypertriglyceridemia in subjects with T2DM [12]. This association was replicated in other large diabetic and non-diabetic population samples [10,13]. Besides the triglyceride levels, the SNP was also associated with lower plasma glucose levels, and with lower risk for the development of T2DM [12,17]. Another SNP of GCKR (rs1260326, P446L) is in strong linkage disequilibrium (r^2 ^= 0.96) with rs780094 according the HapMap II data http://www.hapmap.org. In Danish diabetic twins and in the Dutch population rs1260326 was found to be associated with increased insulin secretion and lower plasma glucose [7,21], and association with elevated triglycerides was also confirmed in different populations [8,9]. By contrast, the rs1260326 allele was found to correlate with metabolic traits, but not with susceptibility for the development of metabolic syndrome in the Scandinavian population [22].

In the current study, we could replicate the previously reported positive associations of the two functional variants of GCKR gene (rs780094 and rs1260326) and triglyceride/glucose metabolism in the Hungarian population; our results confirmed the inverse association with serum triglycerides and plasma glucose levels in T2DM, MS and healthy control subjects.

GCKR competitively inhibits GCK, playing a major role in the regulation of insulin secretion and glycogen metabolism and considered as a potential susceptibility gene for T2DM. In the presence of low glucose concentrations both GCK and GCKR are localized in the nucleus of hepatocytes due to metabolic alterations (higher glucose or fructose concentrations) GCK, but not GCKR, translocates into the cytoplasm [23,24]. Furthermore, GCKR is also play a role in the nuclear-cytoplasmic transport and in the protection against degradation of GCK [25-27]. Animal models have also shown that GCKR also regulates the posttranscriptional expression of GCK. Taken all these data together, it is obvious that functional change in this regulatory protein may considerably influence the glucose metabolism.

In SNP rs1260326 of the GCKR a C/T change results in a proline to leucine substitution in the amino sequence of the encoded protein. This change is very likely to modify the structure of the protein and if this structural alteration is in the binding site of fructose-6-phosphate or fructose-1-phosphate it can influence the function of the protein.

Increased glycolitic flux, downregulated glucose-6-phosphatase and upregulated GCK, phosphofructokinase and fatty acid synthase result in an increased glycogen synthesis and malonil-CoA concentration and an increased VLDL triglyceride production. These metabolic changes might potentially explain the lower plasma glucose and higher triglyceride levels, however the exact mechanism remains to be elucidated [8,28].

Measuring CIMT, as a surrogate marker of cardiovascular disease, is widely used and validated method in both patients with or without T2DM to detect subclinical atherosclerosis, however to predict the relative risk for the development of future cardiovascular diseases is much more difficult and presumes holistic interpretation of the complex interactions between both genetic and clinical factors.

Besides the conventional cardiovascular risk factors as hypertension, high LDL-cholesterol level, low HDL-cholesterol level, hypertriglyceridemia, also the level of advanced glycation endproducts (e.g. N-epsilon-carboxymethyllysine) confer an independent significant risk for cardiovascular diseases and associated with atherosclerotic lesions not only in diabetic but in normoglycemic subjects [29-34].

Common variants in the GCKR gene were referred to be associated also with higher C-reactive protein levels, which is a favorable atherosclerotic marker. Here we also investigated the lipid traits and the CIMT, which was proved an independent preclinical marker of atherosclerosis and CAD.

## 5. Conclusions

Our results support that rs780094 and rs1260326 functional variants of the GCKR gene are inversely associated with serum triglycerides and fasting plasma glucose levels. As a novel feature, in this report we found, that homozygous rs780094 and rs1260326 GCKR gene variants are associated with CIMT.

## 6. List of abbreviations

BMI: Body mass index; CIMT: Carotid intima-media thickness; CAD; Coronary artery diseases; GCK: Glucokinase; GCKR: Glucokinase regulatory protein; MS: Metabolic syndrome; SNPS: Single nucleotide polymorphisms, T2DM: Type 2 diabetes mellitus.

## 7. Competing interests

The authors declare that they have no competing interests.

## 8. Authors' contributions

MM participated in the design of the study, participated in acquisition of data, prepared and wrote the manuscript, performed the statistical analysis. PK, LJ, AM, VCs, NP, ES and KS carried out the molecular genetic studies. EF carried out the ultrasonography examinations. JCs and KH helped in acquisition of data, IW and BM conceived and coordinated the study. All authors have read and approved the final manuscript.
